# Air Pollution and Birth Outcomes: Health Impact and Economic Value Assessment in Spain

**DOI:** 10.3390/ijerph20032290

**Published:** 2023-01-27

**Authors:** Marcelle Virginia Canto, Mònica Guxens, Anna García-Altés, Maria José López, Marc Marí-Dell’Olmo, Javier García-Pérez, Rebeca Ramis

**Affiliations:** 1 Department of Preventive Medicine, Hospital Central de la Cruz Roja, 28003 Madrid, Spain; 2 Doctoral Program in Biomedical Sciences and Public Health, International Doctorate Program, National University of Distance Education (UNED), 28015 Madrid, Spain; 3 Barcelona Institute of Global Health (ISGlobal), 08003 Barcelona, Spain; 4 Spanish Consortium for Research on Epidemiology and Public Health (CIBERESP), 28029 Madrid, Spain; 5 Department of Medicine and Live Sciences, Universitat Pompeu Fabra, 08002 Barcelona, Spain; 6 Department of Child and Adolescent Psychiatry/Psychology, Erasmus MC, University Medical Centre, 3015 GE Rotterdam, The Netherlands; 7 Departament de Salut, Direcció General de Planificació i Recerca en Salut, 08028 Barcelona, Spain; 8 Institut d’Investigació Biomèdica (IIB Sant Pau), 08003 Barcelona, Spain; 9 Public Health Agency of Barcelona, 08023 Barcelona, Spain; 10 Cancer and Environmental Epidemiology Unit, Chronic Diseases Department, National Centre for Epidemiology, Carlos III Institute of Health, 28029 Madrid, Spain

**Keywords:** low birthweight, small for gestational age, preterm birth, particulate matter, air quality

## Abstract

Air pollution is considered an ongoing major public health and environmental issue around the globe, affecting the most vulnerable, such as pregnant women and fetuses. The aim of this study is to estimate the health impact and economic value on birth outcomes, such as low birthweight (LBW), preterm birth (PTB), small for gestational age (SGA), attributable to a reduction of PM_10_ levels in Spain. Reduction based on four scenarios was implemented: fulfillment of WHO guidelines and EU limits, and an attributable reduction of 15% and 50% in annual PM_10_ levels. Retrospective study on 288,229 live-born singleton children born between 2009–2010, using data from Spain Birth Registry Statistics database, as well as mean PM_10_ mass concentrations. Our finding showed that a decrease in annual exposure to PM_10_ appears to be associated with a decrease in the annual cases of LBW, SGA and PTB, as well as a reduction in hospital cost attributed to been born with LBW. Improving pregnancy outcomes by reducing the number of LBW up to 5% per year, will result in an estimate associated monetary saving of 50,000 to 7,000,000 euros annually. This study agrees with previous literature and highlights the need to implement, and ensure compliance with, stricter policies that regulate the maximum exposure to outdoor PM permitted in Spain, contributing to decreased environmental health risk, especially negative birth outcomes.

## 1. Introduction

Air pollution is considered an ongoing major public health and environmental issue around the globe, affecting urban and rural areas of countries with all levels of income [1]. In 2016, The World Health Organization (WHO) estimated that, approximately 60% of premature deaths attributable to the exposure of air pollution, were associated to particulate matter (PM) outdoor exposure [1,2], and about 13,910 of those deaths corresponded to Spain [3]. Multiple worldwide systematic reviews had documented the association between exposure to air pollution including PM, ozone (O_3_), carbon monoxide (CO), nitrogen dioxide (NO_2_) and sulfur dioxide (SO_2_), and adverse health effects [4,5,6,7,8]. 

Pregnant women and their fetuses are among the most vulnerable groups exposed to air pollution [9]. Some of the pollutants that penetrate the placenta after maternal inhalation and deposit of fine particles in their respiratory tract, have the ability to induce impairing placental function, to decrease transplacental oxygen and nutrient transport, and to produce placental oxidative stress and epigenetics changes, which increases the risk of adverse birth outcomes [10,11]. Several recent meta-analysis and systemic reviews have described the association of long-term exposure to PM during pregnancy and adverse outcomes, such as risk of stillbirth [12,13], low birthweight (LBW) [14,15,16], preterm birth (PTB) [17,18], small for gestational age (SGA) [19,20], congenital anomalies [21,22], and other chronic diseases later on the child’s life [23]. 

The WHO defines LBW as a newborn that weighed less than 2500 g. It has been estimated that more than 20,000,000 infants around the world are born with this condition [24]. This is an indicator of intrauterine growth restriction [25] and could predispose to short-term and long-term adverse health outcomes, including an elevated risk of mortality [26]. Premature birth of a fetus at 37 or less weeks of gestation is considered as PTB. Yearly, 15,000,000 neonates are born with PTB [27], which is also a predisposing factor of neonatal mortality and morbidity, such as cerebral palsy, necrotizing enterocolitis, bronchopulmonary dysplasia and retinopathy of prematurity [28], and is considered as the most common source of death in children before their fifth birthday [27]. 

On the other hand, health impact assessment (HIA) is a methodology that quantify the health effect of implementing public policies [29]. This technique has been applied to estimate the health impacts associated with changes in air quality, showing that decreases in fine PM levels are followed by increases in health benefits [30,31]. In particular, some studies have just assessed the magnitude of health impacts of PM air pollution exposure on several outcomes [32,33,34], but others studies have additionally evaluated the health impacts of reduction of PM concentration using different theoretical policy scenarios [35,36,37,38]. In the Spanish context, the first and nation-wide health impact assessment of air pollution showed that an average annual reduction of 0.7 µg/m^3^ in PM_2.5_ levels would produce a decrease of 1720 premature deaths and an increase of 18% in postpone deaths for year 2011 [39,40]. Focused on birth outcomes, another study analyzed the impact of air pollution and low birthweight in Spain for the period 2001–2009 and showed that 6105 cases of LBW were attributable to PM_10_ exposure [41]. 

Furthermore, health impacts cannot be separated from economic impact and some previous studies have estimated the economic loss attributable to exposure to air pollutant in places such as in China [42], Skopje (North Macedonia) [35] or Tehran (Iran) [43]. In Spain, an assessment of two decades of air pollution policymaking calculated the cost due to PM levels based of disability-related absence of work staff [44]. However, there is not an economical cost assessment based on specific health outcomes such as birth outcomes. 

Therefore, this study aimed to estimate the health impact and the economic value on birth outcomes (LBW at term, SGA, and PTB) attributable to a reduction of PM_10_ levels in Spain. For this purpose, we have established four scenarios: fulfillment of WHO guidelines [45], fulfillment EU limits [46], reduction of the levels across the whole country of 15%, and reduction of 50% as the 1st Spanish National Program for Air Pollution Control (NAPCP) sets [47]. 

## 2. Materials and Methods

### 2.1. Study Period, Population and Study Areas 

We used the information from the Spain Birth Registry Statistics database available from the National Statistics Institute (INE), as well as the mean PM_10_ mass concentrations for every pregnancy. The study population included a total of 288,229 live-born singleton children (defined as a birth of only one child showing signs of life at a gestational age of at least 22 completed weeks or weighting 500 g or more) born between June 2009 and October 2010, from all Spanish territory except Canary Islands, Ceuta and Melilla.

### 2.2. Birth Outcomes

Using the information on birth weight (in grams), infant sex, and gestational age (in weeks) compiled in the Spanish birth registry, we calculated the following outcomes: LBW at term (birthweight <2500 g when gestational age ≥37 weeks), SGA (weight in the lowest 10th percentile for sex and gestational weeks) and PTB (gestational age <37 weeks). In addition, we defined the term “exit LBW” as the number of infants that could have been born without LBW if the reduction of PM_10_ levels were enough to meet the proposed scenarios. 

### 2.3. Air Pollution Exposure Assessment

Spatiotemporal land use random-forest models for PM_10_ were developed with a resolution of 1 km for all Spanish territory except Canary Islands, Ceuta and Melilla. The models combined daily satellite remote sensing data of aerosol optical depth with meteorological parameters (daily mean air temperature, sea-level barometric pressure, precipitations, relative humidity, wind speed and direction, and planetary boundary layer height) and land use variables (geoclimatic zones, resident population, point emission sources, mean elevation, imperviousness surface areas, light at night, land cover types, percentage of vegetation cover, desert dust advection, road density, and proximity to airports, ports, sea, and lakes) [48]. To estimate the PM_10_ levels at the maternal address at child’s delivery, we used a random forest model including several spatial variables such as traffic, land use, and population. All addresses were geocoded within a buffer of 30 m around the house to ensure anonymization. Considering the gestational age of each child and assuming that women did not change residence during pregnancy, we estimated average levels of the entire pregnancy period of each child. We focused on the pregnancy-average air pollution levels because it is the most relevant exposure metric for investigating the association between long-term exposure to air pollution and birth outcomes.

The needed parameters to estimate the health impact came from a parallel analysis within the same project (“Air pollution and birth outcomes: windows of exposure and health and economic impact assessment—the APBO project”) [49,50]. A lineal model between pregnancy-average PM_10_ level and birth weight showed that a reduction of 10 µg/m^3^ of PM_10_ was associated with an increase of 22 g (95%CI = 17.2–28) (linear effect). Logistic models estimated baseline odds ratio (OR) for LBW at term, SGA, and PTB, associated to an increase of 10 µg/m^3^ of PM_10_. The results for LBW at term showed an OR of 1.03 (95%CI = 0.96–1.1), for SGA an OR of 1.05 (95%CI = 1–1.09) and for PTB and OR equal to 1.22 (95%CI = 1.16–1.28). All rates were adjusted for following potential confounding variables: sex of the child, age of the mother, age of the father, marital status, parity, mother’s ethnicity, mother’s education, father’s education, deprivation index, mother and father’s occupation, urbanicity, season of conception, average temperature across pregnancy, and autonomous community of mother’s residence.

### 2.4. Calculation of the Health Impacts

We evaluated the birth outcomes benefits that could be accomplished if PM_10_ exposure was reduced, using two different approaches. First, we used the estimated linear effect. Second, we used the baseline ORs and applied the following standard methodology:

1. Air quality: We computed a baseline scenario and four control scenarios. The baseline scenario was the estimated daily PM levels for 2009–2010 and the four control scenarios were based on:
  - The WHO air quality guidelines: Recommendation of an annual PM_10_ level of 15 µg/m^3^ [43]
  - The air quality standards of EU: Recommendation of an annual PM_10_ level of 40 µg/m^3^ [44]
  - A 15% reduction
  - A 50% reduction

The first two control scenarios were applied to the birth outcomes with PM_10_ levels above the guidelines and the other two scenarios were implied a constant reduction of PM_10_ levels across all cases with our birth outcomes based on the NAPCP [47].

2. Population data: We computed the number of pregnant women by each PM_10_ exposure level and outcome incidence.
3. Birth outcomes effects (OR_o_): We used the association rates of LBW (OR = 1.03), SGA (OR = 1.05) and PTB (OR = 1.22) from the parallel study [49,50].
4. Change in PM_10_ concentration (∆C): Related to each scenario in µg/m^3^.
5. Population attributable fraction (PAF): We estimated PAF attributable to the different scenarios and outcomes using the following formula.
  PAF = 1 − (1/OR), where OR = OR_o_^(∆C/10)^
  For changes associated to ∆C = 5 µg/m^3^, OR = OR_o_^(5/10)^

### 2.5. Economic Evaluation

In order to estimate the economic expenses, we extracted the economic health cost associated to each birth outcome that was listed in the minimum basic dataset (CMBD). This is the register of activity data from hospitals in the Spanish National Health System in 2020 [51]. The register contains information on hospital discharge from all Spanish regions and the cost is a weighted average of the mean cost of all cases with a specific condition, calculated by multiplying the number of cases of each condition by its average cost and dividing by the total number of cases in that region. For the present study, we selected the estimated values of hospitalization associated to attending a neonate born with LBW during the postnatal period, defined as the first 6 to 8 weeks after birth. These costs have a minimum and maximum according to the actions that the newborn needs. Once we identified the potential costs, we multiplied them by the estimated PAF, so we could have a range of costs for birth outcome associated with LBW. 

The result represents the annually reduction of costs of preventing cases at national level attributable to the exposure to PM_10_. Other economic expenses such as indirect cost, costs of pain and suffering, financial costs of long-term complications over the course of life, among others, were not included in this analysis.

## 3. Results

### 3.1. Characteristics of the Study Population

A total of 288,229 births were included, with a mean gestational age of 39 weeks and a mean birth weight of 3246 g. The incidence of LBW at term was 3%, 10% for SGA, and 6% for PTB. Table 1 summarizes the descriptive statistics of gestational, maternal, paternal and newborn characteristics of our population distributed by birth outcomes. 

### 3.2. Descriptive Analysis of Pollutants Concentration

The annual mean concentration of PM_10_ across our population during pregnancy was 23.7 µg/m^3^, with a minimum value of seven µg/m^3^ and a maximum of 62 µg/m^3^. This mean value exceeded the 15 µg/m^3^ target proposed by the WHO Air Quality Guidelines in 2021 (exposed more than 98% of the cases), as well as the previous recommendation (Annual mean of 20 µg/m^3^ in 2005) [44]. We estimated that the highest percentage of birth outcomes (40–42%) occurred in the population exposed to mean levels of 20–24 µg/m^3^ of PM_10_, and only one woman was exposed to a mean level of more than 60 µg/m^3^ of PM_10_ concentration (Table 2).

### 3.3. Potential Benefits of Different Scenarios of Reducing Annual PM_10_ Levels on Birth Outcomes Using the Linear Effect

Between 2009 and 2010, the total number cases of LBW exposed to more than 15 µg/m^3^ of PM_10_ level was 8451 infants. The reduction of PM_10_ levels to meet the WHO recommendation could have prevented an estimated number of 383 neonates with LBW attributable to PM_10_ exposure, translated to a decrease of 4.5% cases of LBW compared to the baseline scenario (Table 3). If evaluated separately by sex, the benefit is just slightly superior in female, preventing 4.71% of the cases of LBW compared to a prevention of 4.29% in males (Table 3). 

Conversely, if the PM_10_ mean concentration levels were limited to the EU recommendation of less than 40 µg/m^3^ for the maximum annual PM_10_ levels of exposure, the number of neonates born with LBW will have not been affected (Table 3), and will have been the same incidence of LBW as the baseline scenario. 

For the third scenario, assuming an annual mean PM_10_ concentration reduction of 15%, we estimated that 30 infants born with LBW at term could be avoided (0.3%) (Table 4). If the benefit is evaluated separately by sex, it was found that female will have a marginally higher number of cases avoided of LBW compared to male (0.38% vs 0.30%) (Table 4). 

For the last and most ambitious scenario, assuming a reduction from the baseline exposure to a target of 50% of PM_10_ concentration, we estimated that 5% of the infants born with LBW could be avoided, translating in the possibility of 424 infants born with healthy birthweight at term (Table 4). If this benefit is analyzed by sex, female newborns would avoid more cases of LBW than male (5.25% vs 4.48%). 

### 3.4. Impact of PM_10_ Levels on Birth Outcomes by Recommended Scenarios Annual PM Levels for LBW, SGA and PTB Using the Baseline ORs

Our results showed that a reduction in PM_10_ exposure levels was associated with a decrease in the odds for LBW. This was particularly significant in the WHO reduction scenario, especially in the groups exposed to the highest PM levels (ORs between 0.88 and 0.92), showing that it could be avoided between 1 to 14% of the cases of LBW if this recommendation were fully followed (Table 5). 

For SGA, the results revealed that there is a decrease in the odds of been born SGA if there were a decrease in PM_10_ exposure during pregnancy, especially if the WHO recommendation is followed as in the case of LBW. Moreover, it was found that 1 to 25% of the cases born been SGA could have been avoided just by following any of the scenario’s recommendations studied (Table 5). 

For PTB, the analysis showed a significant decrease in this odd at all scenarios and at all levels of exposure, and from the three outcomes studied. This outcome was the one that showed more benefit of avoiding cases if all scenarios’ policies are applied (Table 5). 

### 3.5. Economic Impact of Reducing Annual PM_10_ Levels on Birth Outcomes

Infants who were born with weights between 2450 and 2496 g could have been candidates to be born without LBW if the recommendations were applied. Minimum and maximum hospital cost was based on the hospital stay of an infant born with 2000–2499 g with or without additional problems and they ranged between 3257 and 27,554 euros per newborn. The previous estimation was compared to a normal infant born with more than 2499 g, which cost of this hospitalization was estimated between 1588 and 10,476 euros per newborn.

**Figure 1:**
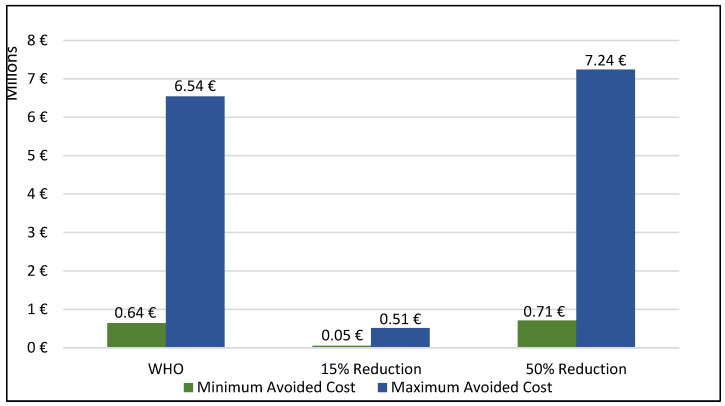
Benefits of PM10 emission reductions. Total avoided hospital cost of the scenarios.

If Spain could diminish the mean levels of PM_10_ by the standards recommended of WHO, approximately 380 neonates would be born without LBW. As well, if a recommendation of a reduction on the mean levels by 15% is reached, 30 neonates would be born without LBW and if a reduction of 50% is applied, approximately 420 neonates would be born without LBW, representing a significant cost saving of up to 7,200,000. Figure 1 represents the monetary benefits of implementing PM_10_ emission reduction policy and the total avoided hospital cost among each recommended scenario based on the health benefits alluded in Table 3 and Table 4. Following the EU scenario did not imply monetary benefits since no cases of LBW would be avoided if this recommendation would be reached.

## 4. Discussion

This study focused on the health and economic impact associated to the exposure to PM_10_ during pregnancy over birth outcomes in Spain, based on meeting the recommendations of WHO or EU, as well as an overall reduction of 15% or 50% PM_10_ levels. Our findings showed that a decrease on the annual exposure to PM_10_ appears to be associated with a decrease in the annual cases of LBW, SGA and PTB, as well as a reduction in hospital cost related to LBW. 

To our knowledge, this is the first study that estimated the health and economic impact of PM_10_ over birth outcomes. Previously, few systematic review and meta-analysis have described the association between PM exposure and negative effects on birth outcomes [12,15,16,52], but neither of them included health and economic impact assessments. Therefore, the present results are not comparable with previous studies. What we can compare is the OR used to compute the PAF. A meta-analysis over 18 studies, found an increment in risk of PTB with increments in 10 µg/m^3^ of PM_2.5_ exposure during the entire pregnancy with a slightly inferior OR to the one used in our study [53]. Another even larger meta-analysis based on 62 studies [54], found very similar computed baselines association rates of LBW as the one that we used. However, the association rate for PTB was remarkably higher compared to ours. In addition, other systematic review of studies conducted between 1980 and 2015, found a negative association between birth weight as well as an incremented risk of PTB [55], with equal OR estimated the ours. Generally, the ORs found in the literature did not differ much from those we used, what means that similar PAFs would have been obtained using the ORs from the meta-analyses and consequently, similar health and economic impact would have been estimated. The only previous study of health impact of PM_10_ on low birth weight in Spain showed a PAF of 9.42% attributable to PM_10_ exposure during pregnancy [41]. Nevertheless, the authors did not carry out a health impact assessment comparing different scenarios. Moreover, they used aggregated data at province level and used weight at birth instead of birth weight at term (gestational age ≥37 weeks). These differences hinder the comparison of the results between the two studies.

In relation to the four different scenarios. The results from the first approach (linear effect) showed that the best scenario would be the overall reduction of PM10 levels by 50%, with a 5% reduction on LBW. The second-best scenario would be compliance with WHO guidelines with a 4.5% reduction. A reduction according to EU guidelines would produce minimum health benefits. The results from the second approach (baseline ORs) showed that the compliance with WHO guidelines (level below 15 µg/m^3^) would provide the higher benefic, presenting a decreased risk of developing LBW up to 14% of the estimated incidence. The estimated value of this economic impact would reach up to 6,500,000. Even if the PM_10_ levels could only be decreased to the previous recommendation (levels below 20 µg/m^3^), it would still represent a significant decrease in LBW incidence. In addition, WHO scenario could have avoided 2–25% of the cases of SGA and 10–100% of the cases of PTB, resulting this guideline in an effective recommendation. On the other hand, the laxer scenario recommended, EU standards (40 µg/m^3^) [46], would have no impact in terms of reducing the number of cases of LBW, neither economic benefits, because very few women were exposed to levels under this levels. This is a clear suggestion that the EU would need more restrictive standards in order to improve air quality to optimal levels that will have an impact on birth outcomes. The results for the last scenario showed that reducing levels to half of the baseline scenario could have a reduction of 5% of LBW, similar to the results obtained when complying with the WHO guidelines. Nevertheless, even a target of reducing a 15% of PM levels would have an impact too.

The economic impact was evaluated taking into consideration the total avoided minimal hospital cost among each recommended scenario, based on the health benefits of being born with a healthy weight. The burden of being born with a birth outcome goes beyond hospital expenses, impacting as well on lifelong health, education, social services and on their families [55]. Neonates born with LBW or PT are more susceptible to be re-hospitalized for several conditions, such a breathing problems or neurological abnormalities, as well as developing learning problems, which could trigger school failure or the need of required additional education assistance. Moreover, other intangible costs associated with taking care of a baby under this situation, should also be taken into account, such as emotional and physical burden on the parents or caregivers, impact on employment, and travel, among others [56]. However, we did not include all these expenses because they rely on the assumption of potential late effects of which we did not have information. On the other hand, we decided to use the costs for year 2020 since we did not have costs for the studied period. This last decision could produce an overestimation of the economic impact for the studied period. Nevertheless, we believed that most recent values were more appropriate to present an up-to-date view of what the savings could represent.

Apart from the constraints on the economic impact estimation already mentioned, this study is not exempt of more potential limitations. A main one is the no inclusion of data on previous maternal health history, substance use, complications or diseases during pregnancy that may affect the newborn’s weight. Moreover, we did not have information about age at pregnancy, maternal lifestyle or data of exposure to other air pollutants, when estimating the OR. Those previous variables could interact with our estimates if they were correlated with both exposure to PM_10_ levels and birth outcome and could potentially bias the results. Another limitation is that we estimated PM_10_ at the home address listed in the birth Registry, without consideration that pregnant women could spent more time of a day in other locations, such as work or could have moved to a different address during any stage of the pregnancy. However, this study contains a large sample of approximately to 288,000 records, over a period of 16 months, from all Spanish territory, except Canary Islands, Ceuta and Melilla, extenuating potential bias that could be present because of the limited information, as well as growing the strength of the estimations of our results.

## 5. Conclusions

In conclusion, these results show that complying with the WHO guidelines or an overall reduction of PM_10_ levels by 50% would improve pregnancy outcomes, with an estimated reduction close to 5% of LBW cases and an estimated associate monetary saving of between 50,000 and 7,000,000 euros annually. Even a reduction of 15% would have health benefits. Moreover, this assessment highlights the need to implement, and ensure compliance with, stricter policies that regulate the maximum exposure to outdoor fine PM_10_ levels permitted in Spain, contributing to decreased environmental health risk—especially negative birth outcomes. European Directive guideline is too permissive. Therefore, more restrictive guidelines, aimed at a maximum reduction in air pollution, should be a priority for public health policies in the EU.

## Figures and Tables

**Table 1:** Birth outcomes by gestational, maternal, paternal and newborn characteristics, 2009–2010.

	Total Births	LBW	SGA	PTB
Characteristics (*n*, %) *	*n*	*n* (%)	*n* (%)	*n* (%)
	288,229	8611 (3%)	28,612 (10%)	16,764 (6%)
**Newborn variables**				
Gestation age at birth, mean (weeks)	39.1	38.4	39.2	34.3
Birth weight, mean (grams)	3246	2245	2531	2419
Infant sex				
Male	148,889	3636 (2%)	14,889 (7%)	9315 (4%)
Female	139,340	4975 (2%)	13,723 (6%)	7449 (3%)
**Maternal variables**				
Maternal age, mean (years)	31.7	31.6	31.5	31.7
Maternal age range				
<20	9011	336 (4%)	1069 (12%)	740 (8%)
20–34	192,644	5685 (3%)	19,185 (10%)	10,657 (6%)
35–40	78,609	2301 (3%)	7513 (10%)	4699 (6%)
>40	7965	289 (4%)	845 (11%)	668 (8%)
Maternal autonomous community of residence				
Andalusia	56,085	1714 (3%)	5579 (10%)	3347 (6%)
Aragon	7765	242 (3%)	793 (10%)	463 (6%)
Asturias	5169	181 (4%)	554 (11%)	336 (7%)
Balearic Islands	8244	209 (3%)	726 (9%)	467 (6%)
Basque Country	17,847	403 (2%)	1490 (8%)	799 (4%)
Cantabria	3274	94 (3%)	313 (10%)	174 (5%)
Castile and Leon	13,695	486 (4%)	1589 (12%)	777 (6%)
Castilla-La Mancha	13,395	422 (3%)	1415 (11%)	847 (6%)
Catalonia	48,879	1469 (3%)	4824 (10%)	2744 (6%)
Community of Madrid	41,853	1332 (3%)	4431 (11%)	2628 (6%)
Extremadura	5638	202 (4%)	608 (11%)	317 (6%)
Galicia	13,641	444 (3%)	1370 (10%)	775 (6%)
La Rioja	2439	73 (3%)	247 (10%)	119 (5%)
Navarre	5385	119 (2%)	421 (8%)	281 (5%)
Region of Murcia	10,829	310 (3%)	947 (9%)	596 (6%)
Valencian Community	34,091	911 (3%)	3305 (10%)	2094 (6%)
Maternal size of municipality or capital of residence				
Equal or less than 10,000 inhabitants	42,853	1239 (3%)	4279 (10%)	2403 (6%)
10,001–20,000 inhabitants	29,202	799 (3%)	2777 (10%)	1538 (5%)
20,001–50,000 inhabitants	44,142	1319 (3%)	4406 (10%)	2469 (6%)
50,001–100,000 inhabitants	30,235	905 (3%)	3036 (10%)	1772 (6%)
More than 100,000 inhabitants	29,642	1010 (3%)	3193 (11%)	1758 (6%)
Capital of the province	112,155	3339 (3%)	10,921 (10%)	6824 (6%)
Maternal educational level				
Primary or less	31,309	1162 (4%)	3644 (12%)	2391 (8%)
Secondary	64,315	2232 (3%)	7178 (11%)	4071 (6%)
Post-secondary	86,737	2559 (3%)	8715 (10%)	5019 (6%)
University or more	105,868	2658 (3%)	9075 (9%)	5283 (5%)
Maternal occupation				
Managers	10,709	274 (3%)	941 (9%)	540 (5%)
Technicians	69,455	1763 (3%)	6136 (9%)	3515 (5%)
Skilled manual/non manual	124,433	3800 (3%)	12,737 (10%)	6926 (6%)
Semi-skilled/Unskilled	20,627	679 (3%)	2270 (11%)	1365 (7%)
Homemakers	55,914	1855 (3%)	5790 (10%)	3849 (7%)
Others	7091	240 (3%)	738 (10%)	569 (8%)
**Paternal variables**				
Paternal age, mean (years)	34.0	33.9	33.7	34.1
Paternal educational level				
Primary or less	38,125	1394 (4%)	4426 (12%)	2720 (7%)
Secondary	80,147	2692 (3%)	8658 (11%)	4812 (6%)
Post-secondary	93,403	2634 (3%)	9121 (10%)	5343 (6%)
University or more	76,554	1891 (2%)	6407 (8%)	3889 (5%)
Paternal occupation				
Managers	17,400	439 (3%)	1410 (8%)	962 (6%)
Technicians	69,613	1803 (3%)	6172 (9%)	3756 (5%)
Skilled manual/non manual	122,992	3936 (3%)	12,876 (10%)	7272 (6%)
Semi-skilled/Unskilled	66,233	2122 (3%)	7061 (11%)	4075 (6%)
Others	11,991	311 (3%)	1093 (9%)	699 (6%)

* Mean was used for continuous variables and count (%) were used for categorical variables. LBW: Low birthweight at term. SGA: Small for gestational age. PTB: Preterm birth.

**Table 2:** Birth outcomes by mean level of exposure to PM_10_ during pregnancy, 2009–2010.

Exposure to PM_10_ Levels	Total Births (%)	LBW	SGA	PTB
	288,229	8611	28,612	16,764
5–9 µg/m^3^	111 (0.04)	3	13	8
10–14 µg/m^3^	4897 (1.7)	157	514	272
15–19 µg/m^3^	50,967 (17.7)	1472	5081	2805
20–24 µg/m^3^	123,601 (42.9)	3617	12,112	6745
25–29 µg/m^3^	90,474 (31.4)	2847	9110	5702
30–34 µg/m^3^	15,388 (5.3)	447	1520	1029
35–39 µg/m^3^	2276 (0.8)	55	215	164
40–44 µg/m^3^	323 (0.1)	7	28	29
45–49 µg/m^3^	100 (0.03)	2	7	4
50–54 µg/m^3^	54 (0.02)	3	8	4
55–59 µg/m^3^	37 (0.01)	1	4	2
60–64 µg/m^3^	1 (0.00)	0	0	0

LBW: Low birthweight at term. SGA: Small for gestational age. PTB: Preterm birth.

**Table 3:** LBW by Infant sex and exposure to PM_10_ levels with recommendation of WHO to reduce annual PM_10_ AQG level to less than 15 µg/m^3^.

	Exposure to PM_10_ Levels	Total Birth with LBW by PM_10_	Number of Infants that Exit ** LBW	Portion of Infants that Exit LBW
**Female**	15–19 µg/m^3^	859	3 (3–36)	0.3% (0–4%)
	20–24 µg/m^3^	2108	73 (66–168)	3% (2–8%)
	25–29 µg/m^3^	1624	115 (59–183)	7% (4–11%)
	30–34 µg/m^3^	259	35 (25–56)	14% (10–22%)
	35–39 µg/m^3^	30	4 (2–6)	13% (7–20%)
	40–44 µg/m^3^	3	0 (0–0)	0% (0–0%)
	45–49 µg/m^3^	2	0 (0–0)	0% (0–0%)
	50–54 µg/m^3^	1	0 (0–0)	0% (0–0%)
	55–59 µg/m^3^	0	0 (0–0)	0% (0–0%)
**Male**	15–19 µg/m^3^	613	4 (4–24)	1% (1–4%)
	20–24 µg/m^3^	1509	39 (37–99)	3% (2–7%)
	25–29 µg/m^3^	1223	85 (44–133)	7% (4–11%)
	30–34 µg/m^3^	188	20 (10–34)	11% (5–18%)
	35–39 µg/m^3^	25	2 (2–3)	8% (8.12%)
	40–44 µg/m^3^	4	1 (1–1)	25% (25–25%)
	45–49 µg/m^3^	0	0 (0–0)	0% (0–0%)
	50–54 µg/m^3^	2	1 (1–1)	50% (50–50%)
	55–59 µg/m^3^	1	1 (1–1)	100% (100–100%)
**Total**	15–59 µg/m^3^	8451	383 (258–745)	4.5% (3–9%)
Total *	20–59 µg/m^3^	6979	376 (248–685)	5% (4–10%)

LBW: Low birthweight (<2500 g) at term. * Total based on the previous recommendation by WHO to reduce annual PM10 AQG levels to less than 20 µg/m3. ** Exit: Departure from the condition of LBW.

**Table 4:** LBW by Infant sex and exposure to PM_10_ levels with a scenario of a reduction of 15% of the annual PM_10_ AQG level.

			Reduction of 15%		Reduction of 50%	
	Exposure to PM_10_ Levels	Total Birth with LBW by PM_10_	Number of Infants that Exit * LBW	Portion of Infants that Exit LBW	Number of Infants that Exit * LBW	Portion of Infants that Exit LBW
**Female**	5–9 µg/m^3^	2	0 (0–0)	0%	0 (0–0)	0%
	10–14 µg/m^3^	87	0 (0–0)	0%	4 (0–4)	5% (0–5%)
	15–19 µg/m^3^	859	3 (3–3)	0.30%	43 (36–84)	5% (4–10%)
	20–24 µg/m^3^	2108	8 (8–8)	0.40%	73 (66–161)	4% (3–8%)
	25–29 µg/m^3^	1624	7 (7–7)	0.40%	115 (59–121)	7% (4–7%)
	30–34 µg/m^3^	259	1 (1–1)	0.40%	24 (12–25)	10% (5–10%)
	35–39 µg/m^3^	30	0 (0–0)	0%	2 (1–2)	7% (3–7%)
	40–44 µg/m^3^	3	0 (0–0)	0%	0 (0–0)	0% (0–0%)
	45–49 µg/m^3^	2	0 (0–0)	0%	0 (0–0)	0% (0–0%)
	50–54 µg/m^3^	1	0 (0–0)	0%	0 (0–0)	0% (0–0%)
	55–59 µg/m^3^	0	0 (0–0)	0%	0 (0–0)	0% (0–0%)
**Male**	5–9 µg/m^3^	1	0 (0–0)	0%	0 (0–0)	0% (0–0%)
	10–14 µg/m^3^	70	0 (0–0)	0%	1 (0–1)	1% (0–0%)
	15–19 µg/m^3^	613	4 (4–4)	1%	26 (24–53)	4% (4–9%)
	20–24 µg/m^3^	1509	5 (5–5)	0.30%	39 (37–94)	3% (2–6%)
	25–29 µg/m^3^	1223	2 (2–2)	0.20%	84 (44–88)	7% (4–7%)
	30–34 µg/m^3^	188	0 (0–0)	0%	8 (5–10)	4% (3–5%)
	35–39 µg/m^3^	25	0 (0–0)	0%	2 (0–2)	8% (0–8%)
	40–44 µg/m^3^	4	0 (0–0)	0%	1 (0–1)	25% (0–25%)
	45–49 µg/m^3^	0	0 (0–0)	0%	0 (0–0)	0% (0–0%)
	50–54 µg/m^3^	2	0 (0–0)	0%	0 (0–0)	0% (0–0%)
	55–59 µg/m^3^	1	0 (0–0)	0%	1 (0–1)	100% (0–100%)
**Total**	5–59 µg/m^3^	8611	30 (30–30)	0.30%	424 (284–647)	5% (3–8%)

LBW: Low birthweight (<2500 g) at term. * Exit: Departure from the condition of LBW.

**Table 5:** Potential benefits of reducing annual PM_10_ levels on birth outcomes.

PM_10_ Ambient Concentrations	Improvement in Air Quality	LBW		SGA		PTB	
	∆C/10	OR	Portion Avoided	OR	Portion Avoided	OR	Portion Avoided
**Exposure to PM_10_ levels at WHO scenario (≤15 µg/m^3^)**							
15–19 µg/m^3^	−0.50	0.99 (0.95–1.02)	1% (−2–5%)	0.98 (0.96–1.00)	2% (0–44%)	0.91 (0.88–0.93)	10% (8–13%)
20–24 µg/m^3^	−1.00	0.97 (0.91–1.04)	3% (−4–10%)	0.95 (0.92–1.00)	5% (0–21%)	0.82 (0.78–0.86)	22% (16–28%)
25–29 µg/m^3^	−1.50	0.96 (0.87–1.06)	5% (−6–15%)	0.93 (0.88–1.00)	8% (0–10%)	0.74 (0.69–0.80)	35% (25–45%)
30–34 µg/m^3^	−2.00	0.94 (0.83–1.09)	6% (−8–21%)	0.91 (0.84–1.00)	10% (0–3%)	0.67 (0.61–0.74)	49% (35–64%)
35–39 µg/m^3^	−2.50	0.93 (0.79–1.11)	8% (−10–27%)	0.89 (0.81–1.00)	13% (0–3%)	0.61 (0.54–0.69)	64% (45–85%)
40–44 µg/m^3^	−3.00	0.92 (0.75–1.13)	9% (−12–33%)	0.86 (0.77–1.00)	16% (0–4%)	0.55 (0.48–0.64)	82% (56–100%)
45–49 µg/m^3^	−3.50	0.90 (0.72–1.15)	11% (−13–40%)	0.84 (0.74–1.00)	19% (0–9%)	0.50 (0.42–0.59)	100% (68–100%)
50–54 µg/m^3^	−4.00	0.89 (0.68–1.18)	13% (−15–46%)	0.82 (0.71–1.00)	22% (0–47%)	0.45 (0.37–0.55)	100% (81–100%)
55–59 µg/m^3^	−4.50	0.88 (0.65–1.20)	14% (−17–54%)	0.80 (0.68–1.00)	25% (0–24%)	0.41 (0.33–0.51)	100% (95–100%)
**Exposure to PM_10_ levels at EU scenario (≤40 µg/m^3^)**							
40–44 µg/m^3^	−0.50	0.99 (0.95–1.02)	1% (−2–5%)	0.98 (0.96–1.00)	2% (0–4%)	0.91 (0.88–0.93)	10% (8–13%)
45–49 µg/m^3^	−1.00	0.97 (0.91–1.04)	3% (−4–10%)	0.95 (0.92–1.00)	5% (0–9%)	0.82 (0.78–0.86)	22% (16–28%)
50–54 µg/m^3^	−1.50	0.96 (0.87–1.06)	5% (−6–15%)	0.93 (0.88–1.00)	8% (0–14%)	0.74 (0.69–0.80)	35% (25–45%)
55–59 µg/m^3^	−2.00	0.94 (0.83–1.09)	6% (−8–21%)	0.91 (0.84–1.00)	10% (0–19%)	0.67 (0.61–0.74)	49% (35–64%)
**Exposure to PM_10_ levels at 15% reduction**							
5–9 µg/m^3^	−0.14	1.00 (0.99–1.01)	0% (−1–1%)	0.99 (0.99–1.00)	1% (0–1%)	0.97 (0.97–0.98)	3% (2–3%)
10–14 µg/m^3^	−0.21	0.99 (0.98–1.01)	1% (−1–2%)	0.99 (0.98–1.00)	1% (0–2%)	0.96 (0.95–0.97)	4% (3–5%)
15–19 µg/m^3^	−0.29	0.99 (0.97–1.01)	1% (−1–3%)	0.99 (0.98–1.00)	1% (0–2%)	0.94 (0.93–0.96)	6% (4–7%)
20–24 µg/m^3^	−0.36	0.99 (0.97–1.01)	1% (−1–3%)	0.98 (0.97–1.00)	2% (0–3%)	0.93 (0.91–0.95)	7% (5–9%)
25–29 µg/m^3^	−0.44	0.99 (0.96–1.02)	1% (−2–4%)	0.98 (0.96–1.00)	2% (0–4%)	0.92 (0.90–0.94)	9% (7–11%)
30–34 µg/m^3^	−0.51	0.99 (0.95–1.02)	2% (−2–5%)	0.98 (0.96–1.00)	3% (0–4%)	0.90 (0.88–0.93)	11% (8–13%)
35–39 µg/m^3^	−0.59	0.98 (0.95–1.02)	2% (−2–6%)	0.97 (0.95–1.00)	3% (0–5%)	0.89 (0.87–0.92)	12% (9–16%)
40–44 µg/m^3^	−0.66	0.98 (0.94–1.03)	2% (−3–6%)	0.97 (0.94–1.00)	3% (0–6%)	0.88 (0.85–0.91)	14% (10–18%)
45–49 µg/m^3^	−0.74	0.98 (0.93–1.03)	2% (−3–7%)	0.96 (0.94–1.00)	4% (0–7%)	0.86 (0.83–0.90)	16% (12–20%)
50–54 µg/m^3^	−0.81	0.98 (0.93–1.03)	2% (−3–8%)	0.96 (0.93–1.00)	4% (0–7%)	0.85 (0.82–0.89)	17% (13–22%)
55–59 µg/m^3^	−0.89	0.97 (0.92–1.04)	3% (−4–9%)	0.96 (0.93–1.00)	4% (0–8%)	0.84 (0.80–0.88)	19% (14–24%)
**Exposure to PM_10_ levels at 50% reduction**							
5–9 µg/m^3^	−0.45	0.99 (0.96–1.02)	1% (−2–4%)	0.98 (0.96–1.00)	2% (0–4%)	0.91 (0.89–0.94)	9% (7–12%)
10–14 µg/m^3^	−0.70	0.98 (0.94–1.03)	2% (−3–7%)	0.97 (0.94–1.00)	3% (0–6%)	0.87 (0.84–0.90)	15% (11–19%)
15–19 µg/m^3^	−0.95	0.97 (0.91–1.04)	3% (−4–9%)	0.95 (0.92–1.00)	5% (0–9%)	0.83 (0.79–0.87)	21% (15–26%)
20–24 µg/m^3^	−1.20	0.97 (0.89–1.05)	4% (−5–12%)	0.94 (0.90–1.00)	6% (0–11%)	0.79 (0.74–0.84)	27% (19–34%)
25–29 µg/m^3^	−1.45	0.96 (0.87–1.06)	4% (−6–15%)	0.93 (0.88–1.00)	7% (0–13%)	0.75 (0.70–0.81)	3% (24–43%)
30–34 µg/m^3^	−1.70	0.95 (0.85–1.07)	5% (−7–18%)	0.92 (0.86–1.00)	9% (0–16%)	0.71 (0.66–0.78)	40% (29–52%)
35–39 µg/m^3^	−1.95	0.94 (0.83–1.08)	6% (−8–20%)	0.91 (0.85–1.00)	10% (0–18%)	0.68 (0.62–0.75)	47% (34–62%)
40–44 µg/m^3^	−2.20	0.94 (0.81–1.09)	7% (−9–23%)	0.90 (0.83–1.00)	11% (0–21%)	0.65 (0.58–0.72)	55% (39–72%)
45–49 µg/m^3^	−2.45	0.93 (0.79–1.11)	8% (−10–26%)	0.89 (0.81–1.00)	13% (0–24%)	0.61 (0.55–0.70)	63% (44–83%)
50–54 µg/m^3^	−2.70	0.92 (0.77–1.12)	8% (−10–29%)	0.88 (0.79–1.00)	14% (0–26%)	0.58 (0.51–0.67)	71% (49–95%)
55–59 µg/m^3^	−2.95	0.92 (0.75–1.13)	9% (−11–32%)	0.87 (0.78–1.00)	15% (0–29%)	0.56 (0.48–0.65)	80% (55–100%)

∆C: Control scenario—baseline scenario. OR: Odds ratio. LBW: Low birthweight at term. SGA: Small for gestational age. PTB: Preterm birth.

## Data Availability

The data presented in this study are available on request from the corresponding author.
